# Brachdactyly Instigated as a Result of Mutation in GDF5 and NOG Genes in Pakistani Population

**DOI:** 10.12669/pjms.341.12885

**Published:** 2018

**Authors:** Samiullah Khan, Muhammad Mudassir, Naqab Khan, Asmatullah Marwat

**Affiliations:** 1 Dr. Samiullah Khan, Ph.D. Gomal Center of Biochemistry and Biotechnology, Gomal University, Dera Ismail Khan, KPK, Pakistan; 2 Mr. Muhammad Mudassir, M. Phil (Scholar). Gomal Center of Biochemistry and Biotechnology, Gomal University, Dera Ismail Khan, KPK, Pakistan; 3 Mr. Naqab Khan, Ph. D (Scholar). Gomal Center of Biochemistry and Biotechnology, Gomal University, Dera Ismail Khan, KPK, Pakistan; 4 Dr. Asmatullah Marwat, Ph.D. Chairman Department of Biochemistry, Quaid-i-Azam University, Islamabad, Pakistan

**Keywords:** Mutation, Genotyping, Pedigree, Linkage analysis, Polymerase Chain Reaction, Polyacrylamide Gel Electrophoresis

## Abstract

**Objectives:**

Brachdactyly a genetic disorder associated with the abnormal development of metacarpals, phalanges or both which results in the shortening of hands and feet. Mutations in the contributing genes has been recognized with the majority of the investigated syndromic form of brachdactyly. The current study was proposed to examine mutation in NOG and GDF5 genes in a Pakistani family.

**Methods:**

Poly Acrylamide Gel Electrophoresis and Polymerase Chain Reaction was used for the genomic screening and linkage analysis to observe the mutation in genes. The samples were collected from Luckki Marwat district, KPK, while the research study was conducted in the department of Biochemistry, Quaid-I-Azam University, Islamabad, Pakistan.

**Results:**

After survey, family was identified with brachdactyly type A2 and investigated a heterozygous arginine to glutamine exchange in the growth demarcation factor 5 in all the victim persons. Different types of skeletal dysplasia resulted due to mutation in the GDF5 genes. Novel GDF5 genes mutations were reported with distinct limb malformation and sequencing of coding region revealed that the mildly affected individuals were heterozygous while the harshly affected individuals were homozygous.

**Conclusion:**

The current study reported the genetic variability and concluded that the Brachdacytyly type A2 and type B2 resulted due to mutation in GDF5 and NOG genes respectively. A new subtype of brachydactyly (BDB2) was instigated as a result of novel mutations in NOG. The mutation has been reported for the first time in Pakistani population and especially in Pushtoon ethnic population.

## INTRODUCTION

Brachdactyly is a medical term which was originated from two Greek words i.e. Brachy means short and dactylos means digits. Brachdactyly most often resulted as an isolated dysmelia (formation of abnormal limbs) which is an inherited dominant trait.

Cartilage originated morphogenetic protein-1 (CDMP) commonly recognized as Growth Differentiating Factor 5 (GDF5), is a predecessor polypeptide having 501 amino acids with seven most conserved cysteine at C-terminal. The GDF5 is in the form of dimer which undergoes proteolysis and producing a mature and dynamic GDF5 dimer which is released by the cell.^1^ GDF5 exhibit an important role in the improvement of joint and skeleton of the embryo.^1^-^3^ GDF5 gene mutation can result various types of skeletal abnormalities including brachdactyly type A2. The congenital abnormalities are associated with either isolated or complex malformation syndrome.^4^

Brachdacytyly may also be associted with others hand dysfunctions such as polydactyly, syndactyly and symphalanges. The international Nosology classified the brachdactyly as a genetic skeletal dysplasias affecting the limbs. The anatomic classification of brachdactyly was provided by the Bell.^4^

Brachdactyly differ from the osteochondrodysplasias as the brachdactyly usually occurs in the blastola stage which is the first eight week of embryonic life while the osteochondrodysplasias usually present in the latter stage of development. Different types of brachdactyly are characterized with different abnormalities such as brachdactyly type A2 which is also known as Mohr-Wriedt type brachdactyly, characterized with short fingers. Brachdactyly type B2 is characterized by aplasia/hypoplasia of the distal phalanges along with the symphalangism, BDB2 is due to missense mutation in NOG gene.^5^

Diagnosis of isolated form of brachdactyly in embryonic stage is not indicated but can be detected in syndromic form, short phalanges cannot be determined by fetal ultrasonography in early developmental stages. The rate of inheritance in offspring of the victim individuals is about 50% irrespective of the gender.^6^

Currently there is no medical treatment of brachdactyly in general, plastic surgery is the only single therapy if hand functions are affecting, physiotherapy and ergotherapy may restore the hand functioning.^7^

Prognosis of the disease mainly depends upon the character of the brachdactyly and may show a discrepancy from affecting hand functioning, prognosis mainly depends upon the nature of characteristics abnormalities. Current reports revealed that joint morphogenesis and multiple synostoses syndrome were caused due to heterozygous mutation of the human noggin gene (NOG).^8^ The current study identified seven mutation of NOG genes from dissimilar families exaggerated with joint morphogenesis.

## METHODS

During survey a family unit of Pushtoon ethnicity was recognized in the distant region of district Lakki Marwat, Khyber Pukhtoonkhwa. The family unit was visited and blood samples were collected along with the complete record of commencement and rigorousness of disease and other abnormalities. Purpose of the present investigation was explained and permission was taken from each individual of the family. The selected individuals were in the range of 3-30 years of age. The ethical approval of the study was obtained from Ethical Review Committee of the Quaid-i-Azam University, Islamabad.

Pedigree was constructed by interviewing the well informed elder member of the ancestors.^9^ Gents persons were indicated by square and female by circle, filled symbols were used to identify affected while unfilled symbols were used to represent normal individuals, crossed square and crossed circle symbols representing the decreased male and female individuals correspondingly. Generation number was indicated by Roman numerals in downward sequence while Arabic numerals from left to right sequence showing persons inside the age group. Tangential blood specimen were taken by an intravenous 5c.c disposable syringe from normal and affected individuals and transferred to a 10ml EDTA tubes and stored at 4°C. Blood samples were subjected for DNA extraction, genomic DNA were extracted by standard phenol-chloroform procedure.^10^

Microsatellite markers of the known loci were used for genotyping and linkage analysis to cause hereditary brachdactyly (Table-I), Genelink invitrogen (USA). The microsatellite markers were amplified by the polymerase chain reaction in 0.2 ml tubes, final volume of the mixture was attuned to 25 µl by the addition of 2µl DNA (Table-II), the mixture was centrifuged 8000 rpm for 30 seconds (T3-Thermocycler, Biometra, Gottingen, Germany).

**Table-I:** List of microsatellite markers used to test linkage to candidate genes/loci.

S. No.	Candidate Gene/Loci	Chromosomal Location	Marker	Distance (cM)*
1	GDF5 Growth Differentiation Factor	20q11.22
			D20S477	51.14
			D20S865	55.42
			D20S478	58.9
			D20S881	58.9
			D20S107	60
2	NOG	17q22
			D17S752	78.41
			D17S787	79.6
			D17S669	82.01
			D17S1853	85.94

**Table-II:** List of chemicals used in PCR.

Chemical	Stock Concentration	Amount used	Final Concentration
PCR Buffer	10 X (200 mM Ammonium persulfate, 750 mM Tris-HCL (pH 8.8), 0.1 % Tween 20)	2.5 µl	1 χ
MgCl2	25Mm	1.5 µl	1.5mM
dNTPs	10mM each dNTPs	0.5 µl	0.2mM
Microsatellite Marker (Reverse)	20ng/µl	0.3 µl	0.24ng/µl
Microsatellite Marker (Forward)	20ng/µl	0.3 µl	o.24ng/µl
Taq Polymerase	0.5U/µl	0.3 µl	0.006U/µl

The PCR was operated for 39 cycles of amplification, each cycle included denaturation of the target DNA at 95°C for one minutes, hybridization of microsatellite markers to the complementary sequence on either side of the target DNA for one minutes at (57-60°C) and last step, expansion of primer was proceeded at 70°C for one minutes and polymerization was occurred at 72°C for ten minutes.

After polymerization, the amplified product was separated on native PAGE (non-denaturing polyacrylamide gel electrophoresis). The gel solution was prepared in 250ml cylindrical flask by mixing 13.5ml 30% acrylamide mix, 5ml TBE and 400µl of 10% APS (Table-III).

**Table-III:** List of chemicals used in PAGE.

Chemicals	Composition and concentration of stock	Amount/gel
30% Acrylamide	29:1 ratio of acrylamide (MERCK, Darmstadt, Germany) N-N Methylene-bisacrylamide (BDH, Poole, England)	13.5 ml
10 x TBE	Tris 0.89 M, Borate 0.89 M and EDTA 0.02 M	5 ml
10% APS	Ammonium persulfate (5 g/45 ml distil water) (Sigma Aldrich St Louis, MO, USA)	400 μl
TEMED	N, N, N', N'-Tetra methyl ethylene diamine) (Sigma-Aldrich, USA)	25 μl
Distilled water		Up to 50 ml

The ultimate volume of the resulting mixture was adjusted to 52ml by adding distilled water, 25µl Tetramethylenediamine (TEMED) was supplemented as a solidifying agent. Gently the solution was mixed in a cylinder, glass plates were used as a spacer having a distance of 1.5 mm apart. The space between the glass plates was filled with the solution, comb was inserted for wells formation and kept for 40 minutes at room temperature. After solidification the gel sandwich was fixed in a vertical gel tank, a running buffer 10X TBE was filled in the gel tank and combs were carefully removed. A loading dye (5µl) was supplemented to each PCR tube having amplified product, the amplified product and loading dye were mixed with stirrer and transferred into the wells.

Electrophoresis was operated for three hours at 120 volts, ethidium bromide was used as detecting dye and gel was placed in (0.5µg/ml) solution of ethidium bromide. The stained gel was visualized by UV trans-illuminator (Biometra, Germany). DC 290 digital camera was used to capture gel pictures.

## RESULTS

**Fig. 1:**
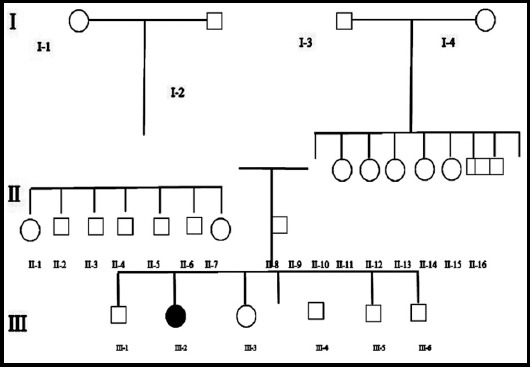
Pedigree of family with autosomal recessive Brachdactyly. Circles and squares represent females and males, respectively. A filled symbol represents an affected individual. The Roman numerals indicate the generation number of the individuals within a pedigree while Arabic numerals indicate their positions within a generation.

**Fig. 2:**
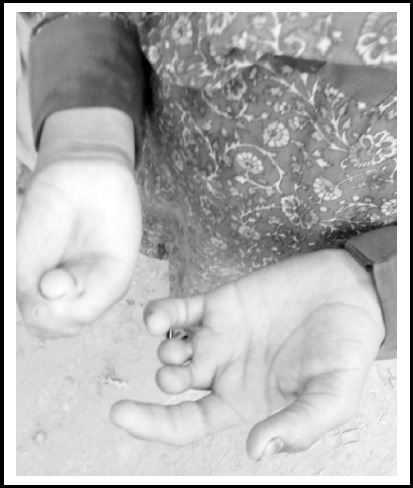
Brachdactyly Affected individual (III-2).

Pedigree was constructed of three generation and 26 individuals (Fig.1), together with one affected character (III-2) as indicated in Fig.2. Blood samples were collected from one affected (III-2) and two normal individuals (II-7 and III-1). Autosomal recessive mode of inheritance was shown by the pedigree as disease appeared in offspring of unaffected parents.

Polymorphic microsatellite markers were used for linkage analysis which were linked to each of the candidate loci/gene (Table-I), to recognize the gene participating in congenital brachdactyly in the selected family.

**Fig. 3:**
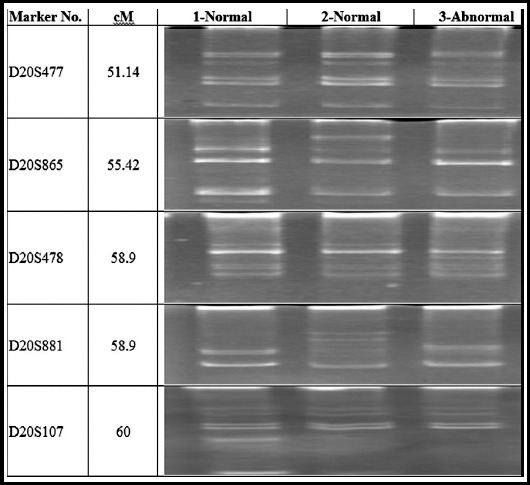
Allelic arrangement achieved with corresponding markers on chromosome 20q11.22 from candidate gene GDF5, shown in electropherogram of ethidium bromide (EtB) stained 8% non-denaturing polyacrylamide gel.

**Fig. 4:**
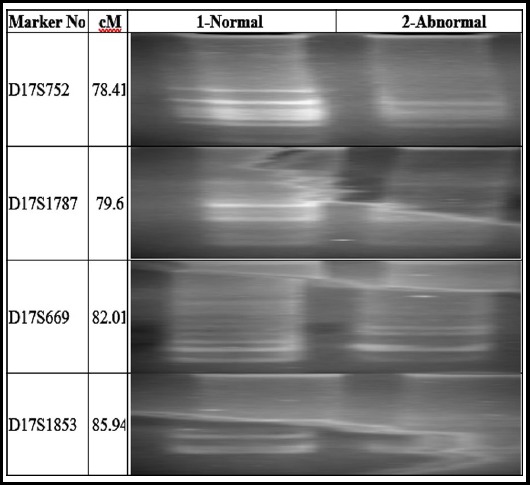
Allelic arrangement achieved with corresponding markers on chromosome 17q22 from candidate gene NOG, shown in electropherogram of ethidium bromide (EtB) stained 8% non-denaturing polyacrylamide gel.

Definite point mutations were investigated in GDF5 and NOG in III-2 (Position in pedigree) associate of the family unit (Fig.3). The extracted DNA samples from one affected (III-2) and two normal individuals (II-7 and III-1) were studied for genotyping, greatly polymorphic microsatellite markers were typed against GDF5 on chromosome 20q11.22 (Fig.3) and NOG on chromosome 17q22 (Fig.4).

Any one of the microsatellite markers did not indicate any linkage to the GDF5 and NOG genes since the affected personage reported heterozygous position at one or two markers of precise gene.

## DISCUSSION

Growth differentiating factor five (GDF5) is an element of bone morphogenetic peptide which is primarily expressed at the site of cartilage demarcation and involved in joint development in limbs.^2^,^3^,^11^,^12^ In developing chicks, the over expression of GDF5 led to fused joints as well as abnormal increase in length and width of whole skeleton.^13^

In the very beginning, GDF5 was investigated in mouse because of its association with brachypodism, phenotypically showed metacarpals, metatarsal, missing middle phalanges and shortened limbs.^14^

Numerous individual skeletal abnormalities characterized by anomalous limb growth are associated with mutation in GDF5 genes, particularly heterozygous mutation in GDF5 which have been known to cause brachdactyly type A2 (BDA2).^5^,^7^,^15^,^16^

Moreover, GDF5 gene mutation are also involved in angel shaped phalangeal dysplasia (ASPED), proximal phalangism (SYM1), multiple synostoses syndrome (SYNS1 and SYNS2) and congenital vertical talus (CVT).^17^-^21^

The P.Cys400Tyr substitution in a consanguineous family, the GDF5 homozygous have chondrodysplasia Grebe type while the heterozygous carriers have brachdactyly phenotypes.^12^ The homozygous and heterozygous phenotypes comparison gives knowledge concerning the type of the anomalous course of embryonic development, leading to inclusive syndrome.^22^

Noggin is a bone morphogenetic protein antagonist and is concerned in the regulation of BMP activity after the initiation of chondrogenesis. In the process of skeletogenesis, the BMPs are concerned in recruiting and demarcation of mesenchymal cells into chondroblasts and osteoblast and stir up program cell death at the site of future joints. As a result of homozygous null mutation due to lacking of noggin, many developmental abnormalities resulted such as missing skeletal elements, shorter bones and wide spread deficiency of articulating joints while the heterozygous mice develop their skeletal structure normally.^23^ Radiological and histological studies of previous work also supports the over expression of noggin in transgenic mice at different ages.^24^ Heterozygous mutation of NOG and GDF5 genes may develop proximal symphalanges which are genetically heterogeneous.^21^ Proximal symphalanges, early onset conductive hearing loss hyperplasia and typical faces are also due to NOG missense mutation.^25^

## CONCLUSION

It is concluded that mutation in GDF5 is the origin of brachdactyly BDA2. The In vitro study investigated that mutant GDF5 was unable to provoke chondrogenesis to the same degree as its wild type counterpart. A novel subtype of brachdactyly (BDB2) is identified as a consequence of new mutation in NOG genes. The mutation has been reported for the first time in Pakistani population and especially in Pushtoon ethnic population. These types of patients are characterized by the occurrence of aplasia/hypoplasia of distal phalanges along with joint fusions.

### Authors; Contributions

***Dr. Samiullah Khan*,** supervisor of research project, provided technical assistance in manuscript write up.

***Muhammad Mudassir*,** research scholar, participated in sampling and PCR operating.

***Naqab Khan*,** helped in designing and composing of the manuscript.

***Dr. Asmatullah Marwat*** co-supervisor provided laboratory facilities and technical assistance.
